# Community Food Insecurity Interventions for Adults Living in the United Kingdom: A Scoping Review

**DOI:** 10.1111/nbu.70026

**Published:** 2025-08-15

**Authors:** Jo Smith, Philip Hodgson, Claire Williams, Amelia A. Lake, Scott B. Teasdale, Emma L. Giles

**Affiliations:** ^1^ Teesside University, School of Health and Life Sciences Middlesbrough, Tees Valley UK; ^2^ Tees, Esk and Wear Valleys NHS Foundation Trust, Flatts Lane Centre Middlesbrough, Tees Valley UK; ^3^ Fuse, The Centre for Translational Research in Public Health Newcastle Upon Tyne UK; ^4^ York St John University York UK; ^5^ Discipline of Psychiatry and Mental Health, School of Clinical Medicine University of New South Wales Sydney New South Wales Australia; ^6^ Mindgardens Neuroscience Network Sydney New South Wales Australia

**Keywords:** food insecurity, food poverty, food security, interventions, scoping review

## Abstract

Food insecurity is a growing concern worldwide, particularly in the United Kingdom. Despite this, community‐based interventions to address food insecurity remain an under‐researched area. Existing food insecurity reviews have focused on international evidence, limiting investigations to foodbank use and/or interventions targeted towards children. This scoping review aimed to understand the evidence on available community‐based interventions for adults experiencing food insecurity in the United Kingdom and the suggested elements for a feasible, acceptable intervention. A comprehensive electronic search was completed up to January 2024. All study designs were considered. A descriptive analytical approach was used to summarise intervention data. Narrative synthesis explored the data further, using the Food Ladders model as a framework. This review identified a very limited scope and quantity of evidence on community food insecurity interventions for UK adults, with 21 included studies. Over half of interventions (52.4%, *n* = 11) relied on volunteers, and a high proportion used donated or surplus food. The nutritional quality of emergency food provision was poor, and it was unclear whether providers could adequately cater for special dietary requirements, cultural and/or religious needs. There were very few studies (19.0%, *n* = 4) assessing the feasibility or acceptability of interventions or their impact on food insecurity. Further research is required into the feasibility, acceptability and effectiveness of community food insecurity interventions for adults in the United Kingdom.

## 1 Introduction

### 1.1 Rationale

Food insecurity is a long‐standing issue worldwide. It was first discussed by the 1974 World Food Conference as being the right to be free from hunger and malnutrition in order to develop one's physical and mental faculties (UN General Assembly 1974). Almost 50 years after the World Food Conference's pledge to eradicate food insecurity within 10 years, it remains a significant worldwide concern (Food and Agriculture Organization of the United Nations 1996). Food insecurity occurs when individuals do not have reliable access to sufficient nutritious food to meet their needs. This includes not only dietary needs but also social and cultural needs (Blake 2019b). In the United Kingdom, 10% of households were experiencing food insecurity between 1 April 2022 and 31 March 2023 (Department for Environment Food and Rural Affairs 2024). The UK's rate of food insecurity is considered amongst the worst in Europe, with this ranging from less than 1% in Cyprus, less than 5% in Sweden and over 10% in Germany up to reported rates of approximately 25% in the United Kingdom and Belgium (Carrillo‐Álvarez 2023). As the global restrictions to manage the Covid‐19 pandemic reduced food access/affordability, the UK's growing hunger crisis increased, with families being driven into poverty and facing a cost‐of‐living crisis (The Food Foundation 2022a). The pandemic exposed the severity of food insecurity in the United Kingdom, and the situation is set to get bleaker as food prices continue to rise. Since the Covid‐19 pandemic, people's reliance on foodbanks has increased rapidly (The Food Foundation 2022b), with the Trussell Trust providing 3.1 million emergency food parcels between April 2023 and March 2024, an increase of 94% over the previous 5 years (The Trussell Trust 2024). The physical and mental health impacts of an individual's diet are well documented (The Mental Health Foundation 2022). UK adults living with food insecurity are known to have a less diverse diet and irregular meal patterns (Shinwell et al. 2022). Consequently, adults experiencing food insecurity in the United Kingdom are more likely to have poor‐quality diets (Keenan et al. 2021). Food insecurity can increase the risk of preventable conditions including hypertension (Stuff et al. 2004), diabetes (Seligman et al. 2007; Seligman et al. 2010) and hyperlipidaemia (Seligman et al. 2010). Furthermore, as a result of a poor‐quality diet, individuals experiencing food insecurity are at risk of being underweight or living with overweight or obesity (Penne and Goedemé 2021). The mental health impacts of food insecurity include considerable stress and anxiety, which can exacerbate pre‐existing mental illnesses or lead to the development of mental ill‐health (Garthwaite et al. 2015; Puddephatt et al. 2020; Thompson et al. 2018; Giles et al. 2023), and may increase the risk of suicidal behaviours (Kaggwa et al. 2023).

Existing reviews and evidence summaries (Blake and Cromwell 2022; Loopstra 2018; Morris and Hitchcock 2022; Oldroyd et al. 2022; Holley and Mason 2019) have identified that UK community‐based food insecurity interventions range from emergency food provision in the form of foodbanks (providing emergency food parcels), food parcels delivered to people's homes or charitable meals (cooked meals provided free of charge or for a nominal fee). Foodbanks are sometimes called food pantries in other countries. Other community food insecurity support includes social supermarkets or surplus food shops (these are also sometimes called food pantries) selling surplus food at discounted prices; nutrition, cookery and/or budgeting education classes; food membership clubs (providing discounted food for a membership fee); mobile food stores (selling discounted food); and/or food voucher schemes (e.g., fruit and vegetable vouchers). Community interventions range greatly between countries in their design, definition and/or delivery. The evidence‐based Food Ladders model developed by Blake (2019a) introduces some context to the vast range of community interventions available to address food insecurity and proposes three levels of community intervention. Rung one (catching) is a starting point for individuals experiencing a crisis in terms of food access. Rung two (capacity building to enable social innovation) involves individuals who may be experiencing difficulties in accessing sufficient affordable food but are not yet in crisis. Rung three (self‐organised community change) includes supporting communities to achieve their goals through self‐organised projects capitalising on local community assets (Blake 2019a).

This scoping review was designed as a prerequisite to a Delphi study co‐creating a new community‐based food insecurity intervention for adults living with severe mental illness in the United Kingdom (Smith et al. 2024). Despite the growing body of evidence regarding the increasing rate of food insecurity in the United Kingdom, there are currently no UK‐based reviews to understand the breadth and scope of evidence on community interventions to address food insecurity for adults. Published reviews and evidence summaries focus on addressing childhood food insecurity (Holley and Mason 2019); include interventions targeted at children (Morris and Hitchcock 2022; Blake and Cromwell 2022); focus only on foodbanks (Oldroyd et al. 2022) or do not focus solely on the United Kingdom (Loopstra 2018; Oldroyd et al. 2022; Blake and Cromwell 2022). To address this stark health inequality in the UK adult population, some understanding is required into what community‐based interventions are available for adults experiencing food insecurity and whether there is any available evidence about the effectiveness, feasibility or acceptability of community‐based interventions.

### 1.2 Aim and Research Questions

The overarching objective of this scoping review was to understand what evidence is available regarding community‐based interventions for adults experiencing food insecurity in the United Kingdom, and the intervention elements that appear integral for a feasible and effective food insecurity intervention. The research questions were:

1. What is the available evidence on community food insecurity interventions for adults experiencing food insecurity in the United Kingdom?
  - What was the approach used (using the Template for Intervention Description and Replication [TIDieR] checklist [Hoffmann et al. 2016])?
  - Did the intervention appear feasible to deliver and acceptable to end users and/or the individuals delivering it?
  - Did the intervention appear effective in reducing food insecurity and/or improving diet quality?
2. What intervention elements appear integral for a feasible and effective intervention from the available evidence?

## 2 Methods

### 2.1 Protocol and Registration

A scoping review was selected to understand the breadth and scope of the available evidence in the United Kingdom, rather than to systematically focus on specific outcomes. This scoping review was conducted in accordance with the Joanna Briggs Institute (JBI) methodology for scoping reviews (Peters et al. 2020), and the Preferred Reporting Items for Systematic Reviews and Meta‐Analyses extension for Scoping Reviews (PRISMA‐ScR) checklist (Tricco et al. 2018). The PRISMA‐ScR checklist is reported in Table S1. The protocol was published on the Open Science Framework (Smith et al. 2025), and registered as a pre‐registration data collection study. An updated protocol version was added to Open Science Framework prior to publication to reflect some planned deviation from the initial protocol. There was a deviation from this protocol in that the authors later included conference abstracts and non‐peer‐reviewed studies published in the grey literature in addition to peer‐reviewed journal articles. This was due to the very small quantity and scope of peer‐reviewed studies identified from initial database and register searches. Whilst the inclusion of conference abstracts and non‐peer‐reviewed studies (such as impact reports) may compromise the overall quality of the included evidence, it was agreed by all authors that the benefits of including a greater breadth of evidence outweighed the quality‐related risks for this particular scoping review.

### 2.2 Eligibility Criteria

The inclusion criteria are reported in line with PICO guidance (Methley et al. 2014). Population: adults (≥ 18 years) experiencing food insecurity. Participants included individuals identified as having food insecurity by health and social care settings; the voluntary and community sector; local authorities; or other agencies. To identify all possible available evidence, the review also included individuals who self‐identified or self‐presented as having food insecurity (not measured using a validated tool). Intervention: a broad range of community‐based interventions was included targeting individuals' food access, community level interventions and/or food use (such as capacity building through cookery classes). Interventions delivered at policy or government levels were excluded, as the review was intended to inform the design of a new community intervention for food insecurity. As the new intervention will be delivered in North East England, studies were limited to the United Kingdom (England, Scotland, Wales and Northern Ireland) as the country of origin given the focus on a place‐based approach (Public Health England 2021). Comparators: As this was a scoping review, studies with any comparators and studies without comparators were included. Outcome: The review included studies and reports that addressed the review questions, and took a broad view, identifying studies and reports from a range of organisations that support adults experiencing food insecurity in addition to published peer‐reviewed papers. Therefore, all outcomes relating to interventions were considered including outcomes relating to feasibility, or acceptability of the intervention (e.g., attrition rates, intervention uptake levels or participant opinions). Furthermore, outcomes on the impact of interventions on food insecurity and/or diet quality were included. For the grey literature, outcomes also included any key performance outcome data such as reductions in foodbank use as an indication of the potential impact on participants' food insecurity.

### 2.3 Information Sources

To identify a broad range of evidence, this scoping review considered both experimental and quasi‐experimental study designs including randomised controlled trials, non‐randomised controlled trials, single‐group design studies, interrupted time‐series studies and qualitative studies. In addition, analytical observational studies (regarding community food insecurity interventions) including prospective and retrospective cohort studies, case–control studies and analytical cross‐sectional studies were considered for inclusion. This review also considered descriptive observational study designs on community food insecurity interventions, including case series, individual case reports and descriptive cross‐sectional studies for inclusion. Review articles, meta‐analyses and opinion papers were used to search for additional primary studies but were excluded from data analysis. Conference abstracts and opinion papers were originally to be excluded in the review protocol, but due to the small number of studies identified in initial database searches, data were collated from conference abstracts that reported on community food insecurity interventions. In addition to published research studies, a grey literature search was conducted using a range of sources as outlined below.

### 2.4 Search Strategy

The search strategy aimed to locate published and unpublished studies and reports. The following databases were finally searched: ASSIA, AMED, CINAHL, EMBASE Healthcare Management Information Centre (HMIC), PsychInfo, MEDLINE and Web of Science. The Social Care Institute for Excellence (SCIE) register was also searched due to its relevance to the subject area of food insecurity. The grey literature search included OpenGrey and an advanced Google Search (first 100 hits using ‘Food insecurity interventions for adults in the United Kingdom’). Furthermore, websites of charities supporting adults with food insecurity were searched including the Trussell Trust, the Food Foundation, Sustain, Feeding Britain and the Independent Food Aid Network for any interventional studies, impact reports or case studies providing data relevant to the research questions. Included papers and any identified systematic reviews/meta‐analyses/opinion papers were forward (citation) and backward (references) searched for any additional articles. Keywords were generated by reviewing the literature in the field, as well as consulting with health and social care practitioners and voluntary and community sector staff who worked in the field of food insecurity. Table S2 documents the keywords that were used to search the databases. Database headings (e.g., CINAHL subject terms and MEDLINE Medical Subject Headings [MeSH] terms in MEDLINE) were used to ensure that all possible subheadings were identified to the key words.

### 2.5 Selection of Evidence Sources

**FIGURE 1:**
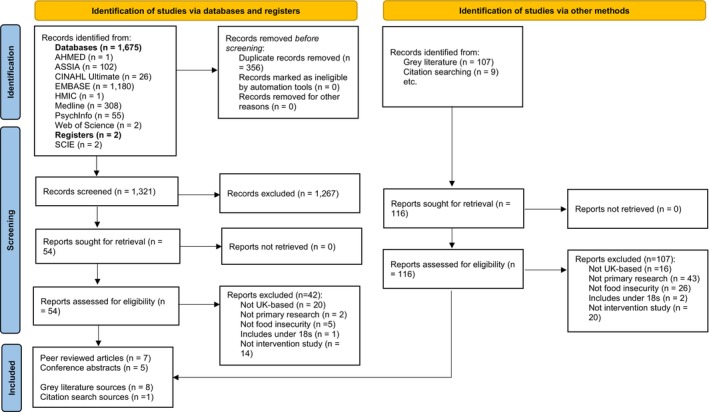
PRISMA 2020 flow diagram. From: Page et al. (2021).

Database and grey literature searches were undertaken from inception to January 2024. All identified citations were collated and uploaded into Rayyan (Ouzzani et al. 2016) and duplicates removed. Titles and abstracts were screened by at least two independent reviewers (JS, PH or CW) for assessment against the review's inclusion criteria. Potentially relevant sources were retrieved in full and assessed in detail against the inclusion criteria by at least two independent reviewers (JS, PH or CW). Reasons for exclusion were recorded on an Excel spreadsheet and are reported in the PRISMA‐ScR flow diagram (Figure 1). Any disagreements were resolved through discussion or by a third reviewer. Approximately 95% of titles and abstracts and full text decisions were reached without requiring a third reviewer. The Hierarchy of Exclusion used to screen articles is outlined in Table S3. Fleiss' kappa (*k*) was used to measure interrater reliability for the full text review (reviewer one and the two independent second reviewers). Fleiss' kappa was 0.899 (95% confidence interval: 0.776–1.022, *p* < 0.001) indicating very good strength of agreement.

### 2.6 Data Charting Process and Items

Data were extracted from papers by two or more independent reviewers using a data extraction tool that was developed and piloted by the reviewers (JS, PH and CW). Data included specific details about the intervention using the TIDieR Checklist (Hoffmann et al. 2016). A summary of the results of the TIDieR Checklist for each study is reported in Tables 1, 2, 3. Further data were extracted outlining the study sampling, sample size, participants' demographic and health information and the main results.

**TABLE 1:** Peer‐reviewed studies published in academic journals (*n* = 7).

Author, date and Food Ladders rung	Study design and population	Intervention details from (TIDieR) Checklist	Variables and measurement tools	Key findings in relation to review questions
Barker et al. (2019) Rung 1	Design: Researcher‐administered cross‐sectional survey and interview Participants: Foodbank attendees Sampling: Purposive Sample size: *n* = 112 Health data: 60% chronic health condition (*n* = 67), with 26% having mental illness (*n* = 29) Mean age: 40.2 (SD: 13.6) years (range 18–72) Gender: 58.9% men (*n* = 66) and 41.1% women (*n* = 46) Ethnicity: White British: 83.9% (*n* = 94); 4.5% African (*n* = 5), 8.1% Asian (*n* = 9), 1.8% European (*n* = 2) and 1.8% Caribbean (*n* = 2)	Name: Foodbank Why: Emergency food Materials: Donated food Procedures: Emergency food parcels Who: Volunteers How: Face to face, individual Where: Three Sheffield city centre foodbanks When: Ongoing How much: Not known, funded by Trussell Trust Tailoring: None Modifications: None	Nutritional quality: 24‐h Multiple Pass Recall, analysed using NETWISP 4.0 Food insecurity: Low Income Diet and Nutrition Survey	Nutritional quality: Diet quality of people using the foodbank was poor, with energy, protein, fibre, iron and calcium intakes inadequate, while saturated fat and sugars intake were disproportionately high Women using the foodbank had poorer diet quality than men Food insecurity: Over half of participants attending the foodbank reported a high level of food insecurity due to lack of money on at least a weekly basis
Fallaize et al. (2020) Rung 1	Design: Cross‐sectional questionnaire and nutritional analysis of food parcels provided by the foodbank Participants: Foodbank managers Sampling: Purposive (one manager for each foodbank) Sample size: *n* = 10 foodbank managers and *n* = 11 food parcels Health data: Not reported Mean age: Not reported Gender: Not reported Ethnicity: Not reported	Name: Foodbank Why: Emergency food Materials: Surplus food and donations Procedures: Emergency food parcels provided by the foodbank Who: Volunteers How: Face to face, individual Where: Ten Oxfordshire foodbanks, including one Trussell Trust foodbank When: Ongoing (parcels covered a 3‐day period) How much: Costs unknown, charity funded Tailoring: Recipe cards, equipment, cooking advice, financial advice and/or toiletries Modifications: None	Nutritional quality: Single user food parcel data were analysed using DIETPLAN7	Nutritional quality: Mean energy, protein, carbohydrate, sugars, fibre and salt content of a parcel were significantly greater than the Dietary Reference Values (DRVs) (*p* < 0.05) and displayed large variance The greatest contributor to energy in the food parcels was carbohydrate (62.2% total energy) For total fat, saturated, poly‐ and monounsaturated fats (g), there were no significant differences between the DRV and parcels, which provided between 87% and 113% of the DRVs With the exception of selenium, significant differences were observed for all other nutrients with the majority providing in excess of the DRVs Retinol and vitamin D were the only micronutrients for which the food parcel did not meet the DRV (27% and 25%, respectively) Food parcels provided 138% of the DRV for energy (may be attributed to the number of days the food parcel is intended to provide intake for) With the exception of copper and vitamin D, for which the Trussell Trust parcel provided significantly less of the nutrient per day than the independent foodbanks (*p* < 0.05), no significant differences were observed between the Trussell Trust and independent foodbank provision
Frost et al. (2016) Rung 1	Design: Mixed methods including ethnography, semi‐structured interviews and nutritional analysis of meals Participants: Charity volunteers Sampling: Purposive sample of volunteers (various roles) Sample size: *n* = 6 Health data: Not reported Mean age: Not reported Gender: Not reported Ethnicity: Not reported	Name: Charity meals Why: Emergency food Materials: Food purchased in supermarkets or ordered online; additional donations received Procedure: Provision of a hot meal (Sunday lunch or weekday breakfast) Who: Charity volunteers How: Face to face self‐service meals with a takeaway option Where: Two charity organisations in Sheffield When: Ongoing, weekly How much: Costs unknown, charity funded Tailoring: None Modifications: None	Nutritional quality: Nutritional analysis of meals using Netwisp Intervention elements: Semi‐structured interviews	Nutritional quality: Meals were adequate for energy and the majority of nutrients, but exceeded thresholds of saturated fat, salt and sugars and lacked vitamin D and selenium in both organisations Intervention elements: *Barriers to success of the intervention* Organisations were constrained by budget, equipment, food donations, volunteer capabilities and time Organisational values influenced meal provision; strategies to reduce fat, salt and sugar content may be resisted because of an ethos of hospitality and overprovision
Garthwaite et al. (2015) Rung 1	Design: Qualitative (Ethnography and semi‐structured interviews) Participants: Adults and volunteers attending a foodbank Sampling: Convenience Sample size: *n* = 42 initial interviews with foodbank users; *n* = 6 follow‐up interviews 2 weeks later; *n* = 8 interviews with volunteers Health data: Not reported Mean age: Foodbank users 18 to 60 years, no data for volunteers Gender: Foodbank users: 20 women and 22 men, no data for volunteers Ethnicity: Not reported	Name: Foodbank Why: Emergency food Materials: Donated food Procedure: Emergency food parcels provided by the foodbank Who: Volunteers How: Face to face, individual Where: Stockton‐on‐Tees town centre When: Ongoing How much: No costs known, funded by the Trussell Trust Tailoring: None Modifications: None	Diet quality: Semi‐structured interviews (users and volunteers) Food insecurity experiences: Semi‐structured interviews (users)	Diet quality: Foodbank users reported having to access poor quality, readily available, filling, processed foods Food insecurity experiences: Foodbank users were on very low incomes—from welfare benefits or insecure, poorly paid employment Many users had pre‐existing health problems which were exacerbated by their poverty and food insecurity Foodbank users were aware of the importance and constitution of a healthy diet, but they were unable to achieve this for financial reasons—constantly having to negotiate their food insecurity
Jennings et al. (2012) Rung 2	Design: Cross‐sectional natural experiment Participants: People from deprived communities with low fruit and vegetable intake and high chronic disease risk Sampling: Convenience Sample size: *n* = 255 Health data: Not reported Mean age: Not reported Gender: 78.4% female (*n* = 200) and 18.8% male (*n* = 55) Ethnicity: Not reported	Name: Mobile food store (MFS) Why: To support deprived communities with low fruit and vegetable intake and high chronic disease risk Materials: Private food wholesaler supplied and maintained a purpose‐built delivery van for use as a MFS, and supplied fruit and vegetables that customers could purchase at wholesale prices Procedure: MFS supplied fruit and veg and promoted positive behaviour change Who: Health Trainers, two members of staff for each van How: Face to face, community Where: Deprived communities in Great Yarmouth and Waveney When: Weekly visits to each area, study duration unknown How much: Costs unknown, study specific funding Tailoring: None Modifications: None	Fruit and vegetable intake: A questionnaire pre‐ and post‐intervention that included a two‐item measure from the East of England Lifestyle Survey (Eastern Region Public Health Observatory, 2008)	Fruit and vegetable intake: Store use resulted in a significant increase in intake of fruit and vegetables (1.2 portions per day, 95% CI 0.83–1.48; *p* < 0.001) The price and the money available to spend on fruit and veg were the most important factors governing respondent's decisions to buy fruit and vegetable
Purdam and Silver (2020) Rung 2	Design: Mixed methods embedded evaluation (semi‐structured interviews) Participants: People on low incomes (including homeless) Sampling: Convenience Sample size: *n* = 108 initial interviews; *n* = 61 follow‐up interviews; *n* = 3 life history interviews; and *n* = 5 interviews with project partners Health data: Not reported Mean age: Not reported Gender: Not reported Ethnicity: Not reported	Name: Cooking and food budgeting classes Why: To help participants develop their cooking and food budgeting skills and so help them reduce the risks of food insecurity Materials: Food provided through a partnership between three foodbank charities, a homeless charity and a food‐sharing charity Procedures: Classes on cookery and food budgeting Who: A trained chef How: Face to face, group setting Where: Four foodbanks in Manchester and Birmingham When: 2‐h sessions (10 participants per session), over 47 weeks How much: Costs unknown, funded by a grant from the local city council Tailoring: None Modifications: None	Intervention elements: Cookery and budgeting confidence and skills assessed through semi‐structured interview, with data analysed in SPSS Food insecurity experiences: Semi‐structured interview questions	Intervention elements: *Knowledge, skills and confidence* Classes had a positive impact on many of the participants, who felt more confident about cooking and valued the shared experience Participants valued learning new cooking skills, sharing their own skills, the information about sauces and spices, and the food budgeting tips 75% of participants felt more confident about cooking healthily on a budget, and 72% of participants stated that their confidence in cooking had either improved or greatly improved Overall, 65% of participants reported that they would eat more healthily, but 26% did not feel they would *Social aspects* Participants valued the communal cooking and eating Food insecurity or access: 77% of participants stated that they could not afford to buy their first choice of food items 23% of participants stated that they had difficulty getting to the shops due to travel costs and mobility issues
Relton et al. (2022) Rung 2	Design: Feasibility study using rapid ethnographic assessment and voucher redemption data Participants: People living in four streets of a peri‐urban area of Barnsley Sampling: Convenience Sample size: *n* = 80 households Health data: Not reported Mean age: Not reported Gender: Not reported Ethnicity: Not reported	Name: Product‐specific vouchers Why: To target areas rather than individual families Materials: £5 vouchers. Redeemable at fruit and vegetable stalls or shops in the town centre, who were re‐imbursed within 48 h by the charity Procedures: Vouchers worth £5 per week Who: Fourteen stakeholder representatives, including the local council and public health team How: Voucher delivery to homes. Redeemed at local fruit and vegetable providers face to face, household level Where: Four streets in a peri‐urban area of Barnsley When: Weekly delivery of vouchers over 10 months How much: Costs unknown. Funded by the Alexander Rose Charity Tailoring: None Modifications: None	Fruit and vegetable intake: Four questions on a survey administered pre‐ and post‐intervention Feasibility: Distribution and redemption voucher logs	Fruit and vegetable intake: There was no change in the proportion of householders who reported eating vegetables once a day or more (52%), but the proportion of householders who reported eating fruit once a day or more increased from 60% at baseline to 76% at 10 months There was little change in response to the ‘eating habits’ main meal question Feasibility: Most eligible households joined the scheme (83%), and 89.3% of vouchers issued were redeemed 69.4% of vouchers were used at the local fruit and vegetable shop and 30.6% were redeemed at the stalls

**TABLE 2:** Peer‐reviewed conference abstracts (*n* = 5).

Author, date publication type and food Ladders rung	Study design and population	Intervention details from TIDieR Checklist	Variables and measurement tools	Key findings in relation to research questions
Harper et al. (2021) Conference abstract published in a journal Rung 2	Design: Feasibility study (single group design) Participants: Adults attending a foodbank Sampling: Convenience Sample size: *n* = 42 (2 drop outs) Health data: Not reported Mean age: 36.4 years (SD: 12.0) Gender: 70% female (*n* = 28); 30% male (*n* = 14) Ethnicity: Not reported	Name: Nutrition education group Why: To deliver a nutrition intervention for foodbank users Materials: Foodbank resources used to prepare a meal Procedure: One hour of nutrition education and 1 h cooking fresh vegetable soup Who: A Registered Dietitian How: Face to face, group setting Where: UK foodbank (city/region unknown) When: Two 2‐h sessions (2 weeks apart) How much: Costs and funding unknown Tailoring: None Modifications: None	Intervention elements: Nutrition knowledge, food choice and confidence were measured using a study‐specific questionnaire (completed pre‐ and post‐intervention) Feasibility: Foodbank user uptake and attendance rates	Intervention elements: *Nutrition knowledge and confidence* More participants knew the recommendations for physical activity (55%) and recognised the Eatwell Guide (40%) after the intervention (*p* < 0.001) Participants knew the recommendations for fruit and vegetable intake pre‐intervention, but their intake was low; 2.4 portions/day Confidence for all variables increased with statistical significance (*p* < 0.001) Feasibility: Attrition from recruitment to attendance was high (42%)
Howard and Green (2021) Conference abstract published in a journal Rung 1	Design: Cross‐sectional questionnaire Participants: Foodbank volunteers Sampling: Convenience Sample size: *n* = 20 (foodbank A = 8, foodbank B = 12) Health data: Not reported Mean age: Not reported Gender: Not reported Ethnicity: Not reported	Name: Foodbank Why: Emergency food Materials: Donated food Procedures: Emergency food parcels Who: Volunteers How: Face to face, individual Where: Two foodbanks in Coventry When: Ongoing How much: Costs unknown, charity funded Tailoring: None Modifications: None	Intervention elements: Participant knowledge of Coeliac Disease and gluten free foods measured using a piloted paper questionnaire	Intervention elements: *Ability to cater for special dietary needs* Overall, the average participant knowledge score of 10.5/20 (range 0–15) was deemed ‘poor’ Only three participants (15%) correctly identified all gluten‐containing grains Volunteers reported to have received no training regarding Coeliac Disease at the foodbank, and five participants specifically asked for training to be provided
Mead et al. (2022) Conference abstract published in a journal Rung 3	Design: Cross‐sectional online survey Participants: UK‐based adults Sampling: Convenience Sample size: *n* = 477 Health data: Not reported Mean age: Not reported Gender: Not reported Ethnicity: Not reported	Name: Urban agriculture Why: To grow food at home or in other urban areas Materials: Seed, water and time to tend to the produce provided by participants Procedure: Home‐grown fruit and vegetables Who: Participant led How: Individual in participants' own home/allotment Where: Liverpool When: Not reported How much: Costs unknown, participant funded Tailoring: None Modifications: None	Food insecurity: Assessed in an online survey Acceptability: Opinions of home food growing measured by an online survey	Food insecurity: Participants who engaged in home food growing had lower levels of food insecurity than those not engaged in home food growing Perceived food insecurity mediated the relationship between home food growing and well‐being Home food growing was associated with less food insecurity, which in turn was associated with better well‐being Acceptability: Opinions of urban agriculture were positive and unchanged compared to pre‐pandemic
Swettenham and Langley‐Evans (2023) Conference abstract published in a journal Rung 2	Design: Patchwork ethnography Participants: People providing or receiving surplus food distribution services Sampling: Convenience Sample size: 70 visitors and 12 volunteers on average per session Health data: Not reported Mean age: Not reported Gender: Not reported Ethnicity: Not reported	Name: Surplus food distribution Why: To prevent food waste by redistributing surplus food Materials: Surplus food Procedure: Redistribution of surplus food at a reduced price Who: Volunteers How: Face to face, individuals visiting the redistribution centre Where: South West London When: Not reported How much: Costs unknown, funded not reported Tailoring: None Modifications: None	Intervention elements: Ethnography and natural conversation recorded by audio or writing	Intervention elements: *Referral systems* Elements contributing to the success of the project included non‐compulsory referral Interaction between end users and health agencies is instrumental in the navigation of food poverty in nested deprivations *Social and community aspects* Community space to socialise was important for a successful intervention There was strong indication of lack of empowerment or belief of visitors accessing the project, in their right or ability to improve their circumstances
Taylor et al. (2023) Conference abstract published in a journal Rung 2	Design: Mixed methods (online/paper survey and semi‐structured interviews) Participants: People accessing three food membership clubs Sampling: Convenience Sample size: Survey (*n* = 97), Interviews (*n* = 12) Health data: Not reported Mean age: Not reported, range 18–65+ years Gender: 71% female (*n* = 69). 24% male. (*n* = 23) 5% missing data (*n* = 5) Ethnicity: 81% White (*n* = 79) and 19% not reported (*n* = 18)	Name: Food membership club Why: To provide food at reduced costs Materials: Not reported Procedure: Food provided at reduced price for a small membership fee Who: Not reported How: Face to face, individual Where: Wessex When: Not reported How much: Costs unknown, funding not reported Tailoring: Intervention also provides budgeting advice, benefits maximisation, and cookery skills Modifications: None	Food insecurity: Modified 6‐item USDA Food Security Survey module Diet quality: Semi‐structured interviews using data from the baseline survey to quantify food insecurity and assess diet quality Acceptability: Semi‐structured interviews	Food insecurity: Food insecurity prevalence was 84% (*n* = 66 out of 79 answers); 41% had low food security (*n* = 30); and 43% (*n* = 32) had very low food security Diet quality: 32% of participants using the food membership club (*n* = 31) rarely or never ate fruit; with 24% (*n* = 23) eating fruit once a day The most common reported frequency of vegetable consumption was 2–3 times a week (*n* = 26, 27%) and 4–6 times a week (*n* = 23, 24%) Acceptability: The clubs were well received, with participants noticing an improvement in their diet and finances

**TABLE 3:** Grey literature (*n* = 9).

Author, date publication type and food Ladders rung	Study design and population	Intervention details from TIDieR checklist	Variables and measurement tools	Key findings in relation to the review questions
Bags of Taste (2019) Impact report Rung 2	Design: Cross‐sectional impact report (survey) Participants: Group attendees (includes vulnerable adults, and people with mental or physical health conditions) Sampling: Convenience Sample size: Unknown Health data: Not reported Mean age: Not reported Gender: Not reported Ethnicity: Not reported	Name: Bags of Taste Why: To support vulnerable adults, and people with mental or physical health conditions Materials: Food purchased using funding provided by local authorities Procedure: Structured cooking and behavioural change programme motivating participants to independently source and cook homemade meals at £1 a head. Ingredients (recipe kit) and mentor provided, with long‐term support package afterwards (social media, videos, etc.) Who: Mentors, background not specified How: Face to face Where: UK‐wide When: Ongoing How much: Costs unknown Funded by local authorities Tailoring: None Modifications: None	Vegetable intake: post‐intervention survey Diet quality: takeaway use measured by post‐intervention survey Intervention elements: various measures from a post‐intervention survey	Fruit and vegetable intake: 38% of participants increased their intake of vegetables Diet quality: 85% of participants attending the group reduced their use of takeaway foods following attendance Intervention elements: *Financial savings* 79% of participants saved money on takeaways and food bills, with the average saving being £1350 per year *Confidence* 38% of participants reported improved confidence in the kitchen *Social aspects* 85% of participants reported that they enjoyed meeting other people
FEAST With Us (2022) Impact Report Rung 2	Design: Cross‐sectional impact report (survey) Participants: Service users Sampling: Not reported Sample size: *n* = 38 Health data: Not reported Mean age: Not reported Gender: Not reported Ethnicity: Not reported	Name: FEAST With Us Why: To support homeless and vulnerable people Materials: Food from surplus food organisations Procedure: Nutritious community meals, including a healthy eating on a budget education programme Who: A Registered Dietitian, chefs, volunteers and fundraisers How: Face to face, group setting (grass roots community‐based programme) Where: North London When: Ongoing How much: Costs unknown, Funding for operational costs from Camden Council Resilience Funding Tailoring: None Modifications: None	Diet quality: Survey questions on the perceived nutritional quality of meal and self‐reported improvements in diet Intervention elements: Survey questions about community and social isolation	Diet quality: 73.0% of participants felt the meals offered were healthy and nutritious 54.0% of participants reported that their diet had improved through attending FEAST Intervention elements: *Social and community aspects* 91.7% of participants said FEAST gave them a sense of community 54.0% of participants felt FEAST made them less socially isolated
FoodCycle (2022) Impact report Rung 2	Design: Cross‐sectional impact report (survey) Participants: Service users Sampling: Not reported Sample size: Not reported Health data: Not reported Mean age: Not reported Gender: Not reported Ethnicity: Not reported	Name: FoodCycle Why: To support people to access healthy meals, warm spaces and target loneliness Materials: Surplus food Procedure: Community café preparing and serving hot meals Who: Volunteers How: Face to face, group Where: UK‐wide When: Ongoing How much: Costs unknown, charity funded with support from corporate partners Tailoring: None Modifications: None	Fruit and vegetable intake: Survey questions Intervention elements: Survey questions on the social aspects	Fruit and vegetable intake: 81% of participants reported that they ate more fruit and vegetables after attending FoodCycle Intervention elements: *Social aspects* 84% of participants made friends 81% of participants felt less lonely
Hughes and Prayogo (2018) Study published on the Trussell Trust website Rung 1	Design: Cross‐sectional (nutritional analysis of food parcels) Participants: Not applicable (food parcels) Sampling: Random sample of parcels from five foodbanks Sample size: *n* = 71 parcels (from 5 foodbanks) Health data: Not applicable Mean age: Not applicable Gender: Not applicable Ethnicity: Not applicable	Name: Foodbank Why: Emergency food Materials: Donated food Procedure: Emergency food parcels provided by foodbank Who: Trussell Trust volunteers How: Face to face, individual Where: Greater London, five foodbanks When: Ongoing (parcels cover a 3‐day period) How much: Costs unknown, funded by the Trussell Trust Tailoring: None Modifications: None	Nutritional quality: Using photographs of food parcels, the weight and variety of products from each parcel were entered into DietPlan 7 software and nutritional analysis undertaken	Nutritional quality: Food parcels met the nutritional requirements for healthy adults over 3 days, for calories, protein, minerals, trace elements and vitamins (with the exception of Vitamin D) Parcels also had acceptable levels of fat; saturated fat; and trans fats The parcels contained levels of sugar and salt that exceed UK Government and international recommendations, and levels of Vitamin D below these recommendations There was a discrepancy between the food parcels provided by the London foodbanks and the hypothetical food parcel created from the standard Trussell Trust national pick list The measured parcels provided greater levels of calories from protein, fats, carbohydrates, saturated fat, sugar, salt and many micronutrients Whilst the hypothetical parcel provided a nutrient content more in line with UK Government and International recommendations, the salt content was still higher than recommended
Loopstra et al. (2019) Study published on the Trussell Trust website Rung 1	Design: Cross‐sectional (Foodbank survey) Participants: Trussell Trust Foodbanks Sampling: Random sample Sample size: *n* = 114 out of 558 foodbanks Health data: Not reported Mean age: Not reported Gender: Not reported Ethnicity: Not reported	Name: Foodbank Why: Emergency food Materials: Donated food Procedure: Emergency food parcels provided by foodbanks Who: Trussell Trust volunteers How: Face to face, individual Where: UK‐wide, 114 foodbanks When: Ongoing intervention, costs unknown, funded by the Trussell Trust Tailoring: Over 60% of Foodbanks offered other services in addition to food parcel distribution Modifications: None	Intervention elements: Telephone survey	Intervention elements: *Referral systems* Over 60% of independent Foodbanks required new clients to have a referral from a third‐party agency but most indicated that exceptions can be made to this rule Almost 40% of foodbanks did not require clients to have referrals Where referrals were received from third‐party agencies, over 75% of Foodbanks reported receiving referrals from local authorities Jobcentre Plus offices were also very commonly reported referral agencies 44% of independent foodbanks imposed no restriction on how often people could receive food parcels, and an additional 17.5% allowed access to food parcels 18 or more times in a 12‐month period Over 30% of foodbanks restricted access to food parcels to six or fewer times in a 12‐month period *Financial and debt advice* Over 60% of foodbanks offered other services in addition to food parcel distribution, including financial advice and debt management
Sutton Council Meals on Wheels (2023) Case study in Sustain (2023), page 9. Rung 1	Design: Case study Participants: People receiving affordable meals on wheels service Sampling: Not reported Sample size: Not reported Health data: Not reported Mean age: Not reported Gender: Not reported Ethnicity: Not reported	Name: Affordable meals on wheels Why: To provide affordable meals on wheels for people who refer themselves into local authority Materials: Food, people and transport provided by the local authority Procedure: Affordable meals delivered to people's homes, public referrals Who: Local authority staff How: Meals delivered to people's homes Where: Sutton, London When: Not reported How much: Costs unknown, funded by the local authority Tailoring: Additional welfare checks undertaken Modifications: None	Intervention elements: Reported in a case study, measurement method not stated	Intervention elements: *Ability to cater for special dietary needs, cultural and/or religious diets* A variety of diets were catered to including vegetarian and coeliac, religious and cultural needs were provided for *Financial aspects* Meals were affordable at around £4 per meal *Referrals* People were able to self‐refer to the local authority
The Bread and Butter Thing (2022) Impact report Rung 2	Design: Cross‐sectional impact report (survey) Participants: Service users Sampling: Convenience Sample size: *n* = 6600 Health data: Not reported Mean age: Not reported Gender: Not reported Ethnicity: Not reported	Name: The Bread‐and‐Butter Thing Why: To support people living in deprived areas Materials: Food provided by Xcess, the independent food redistribution network Procedure: Low‐cost, pre‐packaged weekly shopping, delivered through mobile food clubs Who: Volunteers How: Mobile food clubs in local communities Where: Greater Manchester, Cheshire, Northumberland, Tyne and Wear, County Durham and West Yorkshire (*n* = 81 hubs) When: Ongoing, weekly sessions How much: Costs unknown, funding provided by from Comic Relief, Sainsbury's, The Purslow Trust and Lyons Trust Tailoring: None Modifications: None	Food insecurity: Survey questions about running out of food and foodbank use Fruit and vegetable intake: Survey questions Intervention elements: Survey questions about the community aspects of the intervention	Food insecurity: 77% of participants worried less about running out of food after the intervention Over 14 000 people had been helped to stop or reduce their use of foodbanks Fruit and vegetable intake: 72% of members had better access to fruit and vegetables after the intervention Intervention elements: *Community aspects* 96% of members said the programme was good for their communities
The Company Shop Group (2022) Impact report Rung 3	Design: Cross‐sectional impact report (survey) Participants: Members of the social enterprise Sampling: Not reported Sample size: Not reported Health data: Not reported Mean age: Not reported Gender: Not reported Ethnicity: Not reported	Name: The Company Shop Group Why: To support anyone in the local area get access to discounted food Materials: Surplus food from supermarkets and purchased using income generated by selling other surplus products to members Procedure: Surplus food redistribution through a company shop sold at discounted prices Who: A social enterprise How: Company shops visited by people from local communities Where: UK‐wide When: Ongoing How much: Costs unknown, funding through income generation (social enterprise) Tailoring: None Modifications: None	Intervention elements: Survey questions about financial savings for participants	Intervention elements: *Financial savings* Participants saved on average £212 in the year
The Trussell Trust (2023) Impact Report Rung 1	Design: Cross‐sectional impact report Participants: Foodbank users Sampling: Not reported Sample size: Not reported Health data: Not reported Mean age: Not reported Gender: Not reported Ethnicity: Not reported	Name: The Trussell Trust Why: Emergency food Materials: Donated food Procedure: Emergency food parcels provided by foodbanks Who: Volunteers How: Face to face, individual Where: UK‐wide When: Ongoing How much: Costs unknown, charity funded Tailoring: High quality financial inclusion advice, hardship helpline, food insecurity prevention Modifications: None	Intervention elements: Impacts reported on financial advice and support provided to foodbank users, measurement method unknown	Intervention elements: *Financial advice* Over 37 000 users received additional financial inclusion advice at foodbanks Over 60% of foodbanks were offering high quality, free financial inclusion advice 44 119 people received advice from the hardship helpline (a 39% increase on the previous year) 10 Foodbanks started offering support hubs to help prevent food insecurity, but this was not quantified

### 2.7 Synthesis of the Results

**FIGURE 2:**
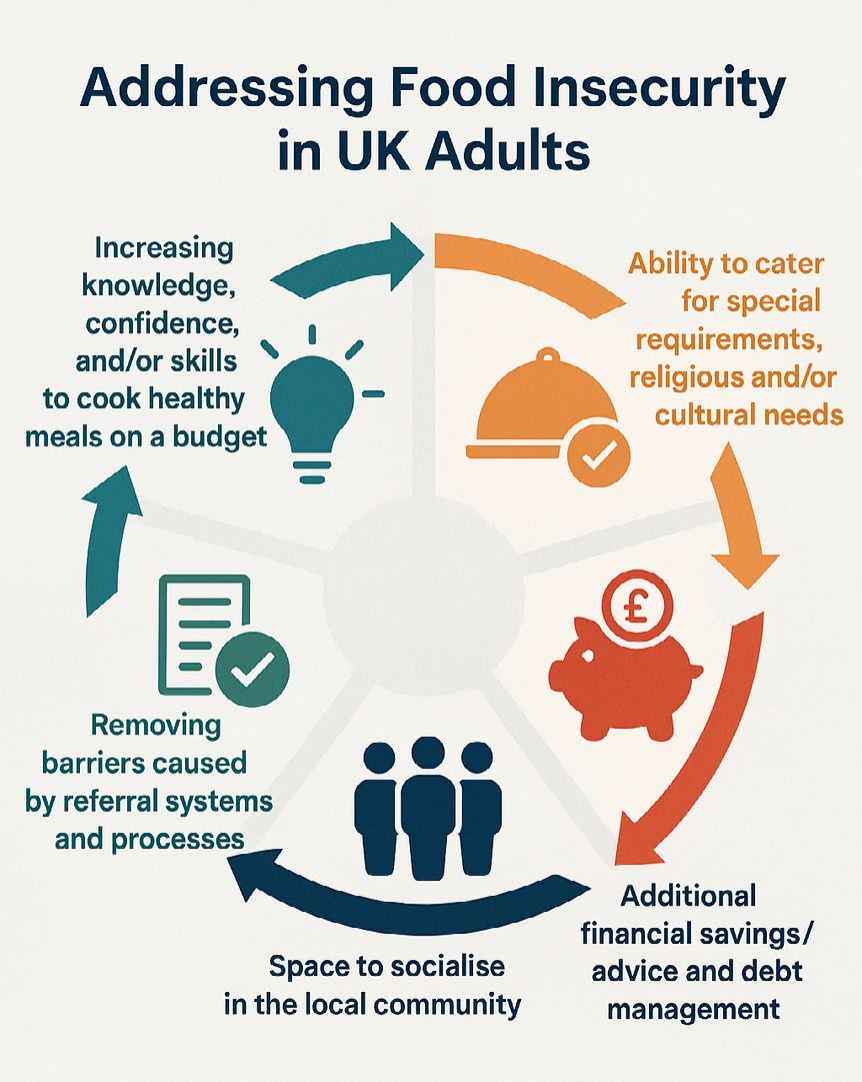
The elements that are important for an effective, feasible and/or acceptable community food insecurity intervention for UK adults.

**FIGURE 3:**
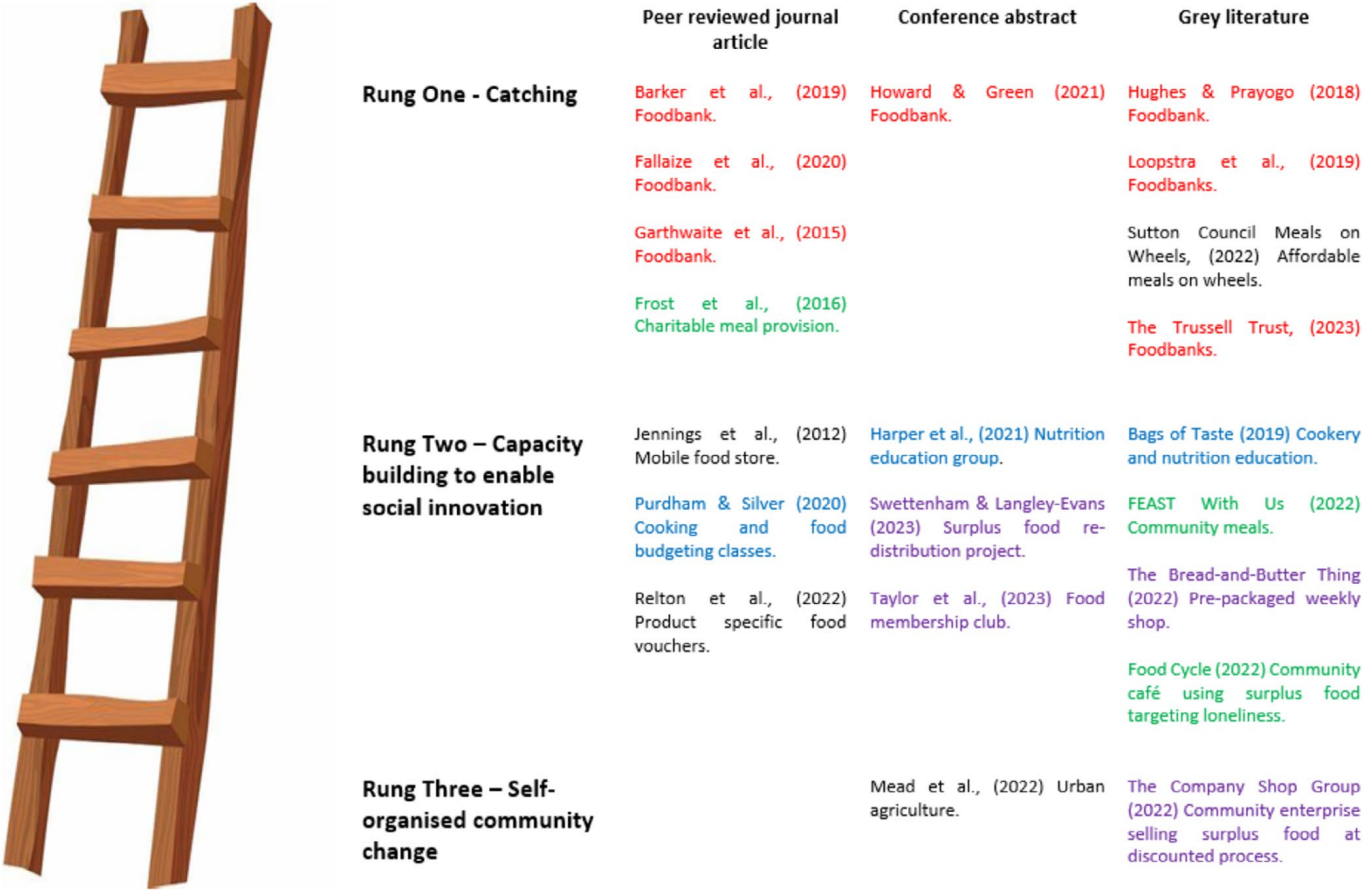
Studies by Food Ladders rung.

A descriptive analytical approach was used to summarise intervention data from the included studies. This aimed to map the key concepts and available evidence, summarise existing research findings and identify gaps in the literature (Pollock et al. 2023). To increase the validity of the results, the analysis was reported in accordance with the Synthesis without Meta‐analysis (SWiM) guideline (Campbell et al. 2020), with data presented in tables and figures where appropriate. Data regarding individual community intervention elements were grouped by commonly reported components (e.g., educational or social elements of interventions) and analysed to understand the potential effectiveness of each component (Figure 2). The Food Ladders developed by Blake (2019a, 2019b) were used to support synthesis of the results, with the interventions being mapped onto each rung of the Food Ladders model (Figure 3). This model was selected as it is the only identified evidence‐based UK conceptual framework that contextualises community‐based food insecurity interventions into levels. The model builds on the United Nations four pillars of food security (availability, access, utilisation and consistency) (Food and Agriculture Organization of the United Nations 2021).

Critical appraisal was not undertaken for this review due to the scarcity of studies reporting data on food insecurity interventions for UK adults identified during the pilot search, and the inclusion of conference abstracts and multiple grey literature sources in this review. Furthermore, critical appraisal was not mandatory for a scoping review (Pollock et al. 2021). Limitations to the study design or evaluation procedure were documented and used to add context around findings during synthesis.

## 3 Results

### 3.1 Selection of Sources of Evidence

The PRISMA flow diagram (Page et al. 2021) is shown in Figure 1.

After removal of duplicates, 1321 citations were identified from databases or registers. Following the title and abstract screen, 54 full text papers were evaluated against the eligibility criteria by JS, PH and CW, and 42 papers were excluded. Seven peer‐reviewed journal articles (Barker et al. 2019; Fallaize et al. 2020; Frost et al. 2016; Garthwaite et al. 2015; Jennings et al. 2012; Purdam and Silver 2020; Relton et al. 2022) and five peer‐reviewed conference abstracts (Harper et al. 2021; Howard and Green 2021; Mead et al. 2022; Swettenham and Langley‐Evans 2023; Taylor et al. 2023) were included in the final data extraction. One hundred and sixteen grey literature sources were screened, and 107 were excluded. Furthermore, nine sources identified via citation searches were screened, with eight being excluded. Eight grey literature reports were included in the final data extraction (Hughes and Prayogo 2018; Sustain 2023; The Trussell Trust 2023; Bags of Taste 2019; FoodCycle 2022; The Bread and Butter Thing 2022; FEAST With Us 2022; The Company Shop Group 2022) and one further grey literature report sourced from citation searching (Loopstra et al. 2019).

### 3.2 Characteristics of Sources of Evidence

#### 3.2.1 Types of Study

Eighty‐one per cent of studies (*n* = 17) were published from 2019 onwards (Barker et al. 2019; Fallaize et al. 2020; FEAST With Us 2022; FoodCycle 2022; Harper et al. 2021; Howard and Green 2021; Loopstra et al. 2019; Mead et al. 2022; Purdam and Silver 2020; Relton et al. 2022; Sustain 2023; Swettenham and Langley‐Evans 2023; Taylor et al. 2023; The Bread and Butter Thing 2022; The Company Shop Group 2022; The Trussell Trust 2023; Bags of Taste 2019), with 19% (*n* = 4) being published in 2023 (Sustain 2023; Swettenham and Langley‐Evans 2023; Taylor et al. 2023; The Trussell Trust 2023). One third of interventions were reported as cross‐sectional studies or impact reports (33.3%, *n* = 7) (Howard and Green 2021; Barker et al. 2019; Fallaize et al. 2020; Mead et al. 2022; Loopstra et al. 2019; Jennings et al. 2012; Taylor et al. 2023). Other study designs were ethnography (19.0%, *n* = 4) (Swettenham and Langley‐Evans 2023; Frost et al. 2016; Garthwaite et al. 2015; Relton et al. 2022), semi‐structured interviews (19.0%, *n* = 4) (Garthwaite et al. 2015; Frost et al. 2016; Taylor et al. 2023; Purdam and Silver 2020), nutritional analysis of food parcels (9.5%, *n* = 2) (Fallaize et al. 2020; Hughes and Prayogo 2018), feasibility studies (9.5%, *n* = 2) (Relton et al. 2022; Harper et al. 2021) and nutritional analysis of charitable meals (4.8%, *n* = 1) (Frost et al. 2016). The remaining interventions were reported as cross‐sectional impact reports (28.6%, *n* = 6) (Bags of Taste 2019; FEAST With Us 2022; FoodCycle 2022; The Bread and Butter Thing 2022; The Company Shop Group 2022; The Trussell Trust 2023) and a case study from a food insecurity report published by Sustain (2023) (4.8%, *n* = 1). Over 47% of studies (47.6%, *n* = 10) used a convenience sample (Garthwaite et al. 2015; Jennings et al. 2012; Purdam and Silver 2020; Relton et al. 2022; Harper et al. 2021; Howard and Green 2021; Swettenham and Langley‐Evans 2023; Taylor et al. 2023; The Bread and Butter Thing 2022; Mead et al. 2022), with purposive sampling being used in 14.3% of studies (*n* = 3) (Barker et al. 2019; Frost et al. 2016; Fallaize et al. 2020). Two further studies used random samples (9.5%) (Hughes and Prayogo 2018; Loopstra et al. 2019). However, five of the impact reports (Bags of Taste 2019; FoodCycle 2022; FEAST With Us 2022; The Company Shop Group 2022; The Trussell Trust 2023) and the case study (Sustain 2023) did not outline the sampling methods used.

#### 3.2.2 Participants

In total there were 8103 participants from studies reporting sample size (66.7%, *n* = 14). Sample size ranged from six to 6600 participants. Four impact reports (Bags of Taste 2019; FoodCycle 2022; The Company Shop Group 2022; The Trussell Trust 2023) and the case study (Sustain 2023) did not outline the sample size used. Only six studies (28.6%) reported the participant sex (Barker et al. 2019; Garthwaite et al. 2015; Harper et al. 2021; Jennings et al. 2012; Taylor et al. 2023), with 66.2% being female (*n* = 363), 32.8% being male (*n* = 180) and 0.9% having missing data (*n* = 5). Two studies (9.5%) reported on participant ethnicity (Taylor et al. 2023; Barker et al. 2019), with 86.5% being white (*n* = 173), 2.5% (*n* = 5) African, 1% (*n* = 2) Caribbean, 1% (*n* = 2) European and 9% missing data (*n* = 18). Age was reported in 19% of studies (*n* = 4) (Barker et al. 2019; Garthwaite et al. 2015; Harper et al. 2021; Taylor et al. 2023), with the age range being 18–72 years. Only one study reported health data for participants, stating that 60% of the sample reported a chronic health condition, with the most common report being related to mental health (26%) (Barker et al. 2019). Two studies (Fallaize et al. 2020; Hughes and Prayogo 2018) reported data on food parcels provided by foodbanks in addition to/instead of participants.

#### 3.2.3 Community Food Insecurity Intervention Approaches

Included studies and reports (*n* = 21) were analysed using the TIDieR checklist to understand the intervention components. The TIDieR components for individual studies are included in Tables 1, 2, 3 (e.g., the materials used, procedures followed and people delivering the intervention).

##### 3.2.3.1 Intervention Types

Community interventions included foodbanks (33%, *n* = 7) (Garthwaite et al. 2015; Fallaize et al. 2020; Barker et al. 2019; Loopstra et al. 2019; Howard and Green 2021; Hughes and Prayogo 2018; The Trussell Trust 2023); education on cooking, nutrition and/or budgeting skills (14.3%, *n* = 3) (Purdam and Silver 2020; Harper et al. 2021; Bags of Taste 2019); community meals providing hot meals at discounted prices (9.5%, *n* = 2) (FEAST With Us 2022; FoodCycle 2022); charitable meal provision for homeless and vulnerable adults (4.8%, *n* = 1) (Frost et al. 2016); an affordable meals‐on‐wheels service (4.8%, *n* = 1) (Sustain 2023); a mobile food store selling low cost fruit and vegetables (4.8%, *n* = 1) (Jennings et al. 2012); product‐specific vouchers for fruit and vegetables delivered to deprived areas (4.8%, *n* = 1) (Relton et al. 2022); a surplus food re‐distribution project (4.8%, *n* = 1) (Swettenham and Langley‐Evans 2023); a low‐cost food membership club (4.8%, *n* = 1) (Taylor et al. 2023); a low‐cost pre‐packed weekly shop (4.8%, *n* = 1) (The Bread and Butter Thing 2022); urban agriculture, with participants growing their own food at home or in other urban areas (4.8%, *n* = 1) (Mead et al. 2022); and a social enterprise re‐distributing surplus food through a ‘Company Shop’ sold at discounted prices (The Company Shop Group 2022).

##### 3.2.3.2 Intervention Locations

Over 28% of interventions were UK‐wide (28.6%, *n* = 6) (Bags of Taste 2019; FoodCycle 2022; Loopstra et al. 2019; The Company Shop Group 2022; The Trussell Trust 2023; The Bread and Butter Thing 2022); others were based in London (19%, *n* = 4) (Swettenham and Langley‐Evans 2023; Sustain 2023; Hughes and Prayogo 2018; FEAST With Us 2022); Yorkshire and the Humber (14.3%, *n* = 3) (Barker et al. 2019; Frost et al. 2016; Relton et al. 2022); the North West (9.5%, *n* = 2) (Purdam and Silver 2020; Mead et al. 2022); the South East (9.5%, *n* = 2) (Jennings et al. 2012; Fallaize et al. 2020); the Midlands (9.5%, *n* = 2) (Purdam and Silver 2020; Howard and Green 2021); the North East (4.8%, *n* = 1) (Garthwaite et al. 2015); the South West (4.8%, *n* = 1) (Taylor et al. 2023); and not stated (4.8%, *n* = 1) (Harper et al. 2021).

##### 3.2.3.3 Intervention Delivery

Over 50% of community interventions were delivered by volunteers (52.4%, *n* = 11) (Barker et al. 2019; Fallaize et al. 2020; Frost et al. 2016; Garthwaite et al. 2015; Howard and Green 2021; Hughes and Prayogo 2018; Loopstra et al. 2019; FEAST With Us 2022; FoodCycle 2022; The Bread and Butter Thing 2022; The Trussell Trust 2023), although one also included paid professionals (FEAST With Us 2022). Other interventions were delivered by Registered Dietitians (9.5%, *n* = 2) (FEAST With Us 2022; Harper et al. 2021); trained chefs (9.5%, *n* = 2) (Purdam and Silver 2020; FEAST With Us 2022); local authorities (9.5%, *n* = 2) (Sustain 2023); health trainers (4.8%, *n* = 1) (Jennings et al. 2012); public health workers (4.8%, *n* = 1) (Relton et al. 2022); mentors (4.8%, *n* = 1) (Bags of Taste 2019); participant‐led (4.8%, *n* = 1) (Mead et al. 2022); a social enterprise (4.8%, *n* = 1) (The Company Shop Group 2022); and not stated (9.5%, *n* = 2) (Taylor et al. 2023; Swettenham and Langley‐Evans 2023).

##### 3.2.3.4 Intervention Resources

Food was provided for interventions from a combination of sources, with the most frequent supplier being donations (38.1%, *n* = 8) (Barker et al. 2019; Garthwaite et al. 2015; Loopstra et al. 2019; The Trussell Trust 2023; Fallaize et al. 2020; Harper et al. 2021; Howard and Green 2021; Hughes and Prayogo 2018). Other sources of food and funding for interventions were surplus food redistribution organisations (19.0%, *n* = 4) (FEAST With Us 2022; FoodCycle 2022; The Bread and Butter Thing 2022; The Company Shop Group 2022); local council funding (14.3%, *n* = 3) (FEAST With Us 2022; Purdam and Silver 2020; Sustain 2023); charity funding (9.5%, *n* = 2) (Frost et al. 2016; The Bread and Butter Thing 2022); a private food wholesaler (4.8%, *n* = 1) (Jennings et al. 2012); local authority funding (4.8%, *n* = 1) (Bags of Taste 2019); fruit and vegetable shops/stalls (reimbursed by a charity voucher scheme) (4.8%, *n* = 1) (Relton et al. 2022); grown by participants (4.8%, *n* = 1) (Mead et al. 2022); income generation in a social enterprise (4.8%, *n* = 1) (The Company Shop Group 2022); and not stated (9.5%, *n* = 2) (Swettenham and Langley‐Evans 2023; Taylor et al. 2023).

### 3.3 Results of Individual Sources of Evidence

The included studies (*n* = 21) were explored to understand the potential effectiveness, feasibility and acceptability of community interventions to address food insecurity in UK adults.

#### 3.3.1 Potential Feasibility and Acceptability of Community Interventions for End Users and/or Those Delivering the Intervention

Nineteen per cent of studies (*n* = 4) assessed feasibility and/or acceptability through a range of outcomes including attrition rates, participant opinions and uptake rates for interventions (Harper et al. 2021; Taylor et al. 2023; Relton et al. 2022; Mead et al. 2022). Harper et al. (2021) assessed the feasibility of a nutrition education group for foodbank users (*n* = 42) and reported that attrition from recruitment to attendance was high at 42%. Taylor et al. (2023) assessed the acceptability of food membership clubs, reporting they were well received with participants (*n* = 12) noticing an improvement in their diet and finances. Mead et al. (2022) assessed the acceptability of home food growing and reported that participants' (*n* = 477) opinions were positive, although they did not quantify the number or percentage. Relton et al. (2022) evaluated the acceptability of a fruit and vegetable voucher scheme, reporting that 83% of eligible households (*n* = 80) joined the scheme, with 89.3% (*n* unknown) of vouchers being redeemed.

#### 3.3.2 The Potential Impact on Food Insecurity

One study (4.8%) attempted to assess the impact of the intervention on food insecurity (The Bread and Butter Thing 2022). The Bread and Butter Thing (2022) helped over 14 000 people to stop or reduce their use of foodbanks and reported that 77% of members (*n* = 6600) worried less about running out of food. However, the authors did not measure food insecurity using a validated food insecurity measurement tool. Furthermore, Mead et al. (2022) reported that participants who engaged in home food growing (*n* = 477) had lower levels of food insecurity than those who were not engaged in this, although they did not quantify the food insecurity levels between the two groups.

#### 3.3.3 The Potential Impact on Nutritional or Diet Quality

Two studies (9.5%) provided data on the nutritional quality of foodbank parcels (Fallaize et al. 2020; Hughes and Prayogo 2018). Fallaize et al. (2020) found that the mean energy, protein, carbohydrate, sugars, fibre and salt content of foodbank parcels (*n* = 11) were significantly greater than the daily recommended intakes for adults (*p* < 0.05). There was wide variation in nutritional quality between different parcels. Hughes and Prayogo (2018) reported that foodbank parcels (*n* = 71) met the nutritional requirements for energy, protein, vitamins and minerals (except vitamin D), and contained acceptable levels of fat, saturated fat and trans‐fats. However, the parcels exceeded daily recommendations for sugar and salt. Two further studies (9.5%) reported on the dietary intake of people attending foodbanks rather than on the impact of foodbanks as an intervention (Barker et al. 2019; Garthwaite et al. 2015). Two studies assessed the nutritional quality of community or charitable meals (Frost et al. 2016; FEAST With Us 2022). Frost et al. (2016) reported that charity meals (*n* = unknown) were adequate for energy and most nutrients, but exceeded thresholds of saturated fat, salt and sugars and were low in vitamin D and selenium compared to daily requirements. Impact reports detailed participants' perceptions of the nutritional quality of the food provided or assessed changes in takeaway use. FEAST With Us (2022) reported that 73% of service users (*n* = 38) felt the meals offered were healthy and nutritious, and 54.0% felt their diet had improved with attendance. Finally, Bags of Taste (2019), a structured cookery and nutrition course supplying recipe kits, reported that 85% of participants (*n =* unknown) reduced their use of takeaway foods.

Six studies (28.6%) measured changes in fruit and/or vegetable intakes (Jennings et al. 2012; Relton et al. 2022; Bags of Taste 2019; Harper et al. 2021; FoodCycle 2022; The Bread and Butter Thing 2022). Jennings et al. (2012) reported that mobile food store use significantly increased participants' (*n* = 255) intakes of fruit and vegetables (mean increase: 1.2 portions per day, 95% CI: 0.83–1.48; *p* < 0.001). Relton et al. (2022) reported that participants (*n* = 80) who received weekly £5 vouchers to purchase fruit and vegetables showed no change in the proportion of participants eating vegetables ≥ once daily (52%), but the proportion eating fruit ≥ once daily increased from 60% at baseline to 76% at 10 months. Harper et al. (2021) reported that participants' (*n* = 42) fruit and vegetable intakes remained low (2.4 portions per day) following a nutrition education intervention within a foodbank. Bags of Taste (2019), a structured cookery and nutrition course with recipe kits, reported that 38% of participants (*n =* unknown) increased their intake of vegetables after attending the intervention. FoodCycle (2022) reported that 81% of participants (*n =* unknown) ate more fruit and vegetables after attending a community café. The Bread and Butter Thing (2022) reported that 72% of mobile food club members (*n* = 6600) had better access to fruit and vegetables after engaging with the intervention. The final study by Taylor et al. (2023) only reported baseline data for fruit and vegetables (daily or weekly frequency of fruit and vegetable intake).

### 3.4 Narrative Synthesis of the Results

The narrative synthesis involved synthesising individual intervention elements and categorising interventions using the Food Ladders model as presented below (Blake 2019a).

#### 3.4.1 Intervention Elements That Appear Integral for a Feasible and Effective Community Food Insecurity Intervention

As shown in Figure 2, the elements that led to feasible and/or acceptable community food insecurity interventions are briefly described below.

##### 3.4.1.1 Increasing Participants' Knowledge, Confidence and/or Skills

Four studies assessed the impact of educational intervention components on participants' self‐reported knowledge, confidence and/or skills to cook healthy meals on a budget (Harper et al. 2021; Purdam and Silver 2020; Bags of Taste 2019; FEAST With Us 2022). All four reported that educational elements of interventions, or stand‐alone educational interventions, had a positive impact on participants' knowledge, confidence and/or skills in cookery, healthy eating and/or budgeting.

##### 3.4.1.2 Providers' Ability to Cater for Special Dietary Requirements, Religious and/or Cultural Needs

Two studies assessed the ability of interventions to cater for participants who had special dietary requirements, religious and/or cultural needs (Howard and Green 2021; Sustain 2023). Whilst Sutton Council Meals on Wheels (2022) simply reported that meals provided met special dietary requirements, religious and/or cultural needs (Sustain 2023), Howard and Green (2021) found that foodbank volunteers had poor knowledge of foods containing gluten and had not received any training on coeliac disease. It is therefore unclear whether providers can adequately cater for a range of special dietary requirements, cultural and/or religious needs.

##### 3.4.1.3 Additional Financial Savings/Advice and Debt Management

Five studies reported data on financial savings, advice and/or debt management services in addition to providing food (The Company Shop Group 2022; Sustain 2023; Bags of Taste 2019; Loopstra et al. 2019; The Trussell Trust 2023). There was a high take‐up of financial elements of interventions offered by the Trussell Trust (The Trussell Trust 2023; Loopstra et al. 2019). Other interventions demonstrated direct financial savings for participants (Bags of Taste 2019; Sustain 2023; The Company Shop Group 2022).

##### 3.4.1.4 Removing Barriers Caused by Referral Systems and Processes

Two studies provided data on referral systems for interventions and some of the barriers (Loopstra et al. 2019) or enablers they presented (Swettenham and Langley‐Evans 2023). It appears that mandatory referral processes may be a barrier to effective interventions, and non‐compulsory referrals (self‐referral or open access) are more likely to contribute to an intervention's success.

##### 3.4.1.5 Space to Socialise in the Local Community

Four studies or reports included data on the social and community aspects of interventions (Swettenham and Langley‐Evans 2023; FEAST With Us 2022; FoodCycle 2022; The Bread and Butter Thing 2022). All indicated that having a dedicated space for participants to socialise, meet new people and feel better connected to their community therefore appears to be an important element for a successful community food insecurity intervention.

#### 3.4.2 Food Ladders Categorisation of the Available Community Food Insecurity Interventions

Figure 3 shows the findings of this review mapped onto the Food Ladders model (Blake 2019a). The coloured text highlights the type of intervention as follows: red = foodbanks (33.3%, *n* = 7); purple = surplus food distribution through social supermarket or social enterprise models (19.0%, *n* = 4); blue = educational interventions (nutrition, cookery and/or budgeting) (14.3%, *n* = 3); gree*n* = community or charitable meal provision (14.3%, *n* = 3); and black = other types of intervention (19.0%, *n* = 4).

Over 42% of interventions (42.9%, *n* = 9) mapped onto rung one, which involves interventions designed to support individuals who are in food insecurity crisis such as foodbanks, food parcels or charitable meals (Barker et al. 2019; Fallaize et al. 2020; Frost et al. 2016; Garthwaite et al. 2015; Howard and Green 2021; Hughes and Prayogo 2018; Loopstra et al. 2019; Sustain 2023; The Trussell Trust 2023). Over 47% of interventions (47.6%, *n* = 10) mapped onto rung two, which involves interventions designed to support individuals who are not in food insecurity crisis but may be struggling to afford sufficient healthy food such as community cafes, healthy cookery and/or budgeting classes, or surplus food redistribution through social supermarkets (Jennings et al. 2012; Purdam and Silver 2020; Relton et al. 2022; Harper et al. 2021; Swettenham and Langley‐Evans 2023; Taylor et al. 2023; The Bread and Butter Thing 2022; FEAST With Us 2022; Bags of Taste 2019; FoodCycle 2022). Approximately 9% (9.5%, *n* = 2) of interventions identified mapped onto rung three of the Food Ladders including urban agriculture and a community enterprise shop selling surplus food (Mead et al. 2022; The Company Shop Group 2022). This rung involves projects that meet a community's needs through self‐organised community change and using community assets, creating safe and inclusive spaces for people to interact with food (Blake 2019a).

## 4 Discussion

### 4.1 Summary of the Evidence and Comparison to Previous Literature

This review identified a limited quantity and scope of evidence regarding a broad range of community interventions to address food insecurity, with a wide variety in the outcomes and measurement tools used. Over half of the included studies (52.4%, *n* = 11) relied on volunteers to deliver the intervention (Barker et al. 2019; Fallaize et al. 2020; Frost et al. 2016; Garthwaite et al. 2015; Howard and Green 2021; Hughes and Prayogo 2018; Loopstra et al. 2019; FoodCycle 2022; The Bread and Butter Thing 2022; The Trussell Trust 2023; FEAST With Us 2022), suggesting that many of the interventions are not sustainable without the volunteer workforce. Furthermore, 42.9% (*n* = 9) relied on donated food (Barker et al. 2019; Fallaize et al. 2020; Garthwaite et al. 2015; Purdam and Silver 2020; Harper et al. 2021; Howard and Green 2021; Hughes and Prayogo 2018; Loopstra et al. 2019; The Trussell Trust 2023) and 28.6% (*n* = 6) used surplus food for the intervention (Fallaize et al. 2020; Swettenham and Langley‐Evans 2023; FEAST With Us 2022; FoodCycle 2022; The Bread and Butter Thing 2022; The Company Shop Group 2022). This further demonstrates the reliance on charity and voluntary sector services to sustain the community‐based food insecurity interventions identified in this review. Over‐reliance on the voluntary workforce and donated food is an important factor influencing interventions' vulnerability to unforeseen events such as the Covid‐19 pandemic (Loopstra and LAMBIE‐Mumford 2023). Donated and surplus food sources are inconsistent in their nutritional quality as they rely on unsold foods from supermarkets and other retailers, or donations from the public or retailers (Mossenson et al. 2024). These results are comparable to those found in other reviews focussed on children's food insecurity or interventions from other countries (Loopstra 2018; Morris and Hitchcock 2022; Holley and Mason 2019; Blake and Cromwell 2022).

The findings of this review indicate that food insecurity interventions may be feasible and acceptable for end users or those delivering the interventions, although reporting of feasibility and acceptability data was limited (*n* = 4 studies). This finding is convergent with an evidence review by Morris and Hitchcock (2022) and an evaluation by Blake and Cromwell (2022), with both only finding one UK study reporting feasibility data for a food voucher scheme (Relton et al. 2022). Feasibility and acceptability data are essential for commissioning of public health interventions (Moore et al. 2018). Given that over 50% of the interventions in this review were delivered by volunteers and that 36.4% of the food was donated, the long‐term sustainability of such interventions is questionable in the absence of feasibility and/or acceptability data.

This review found that a limited number of studies on foodbanks (9.5%, *n* = 2) and charity meals (9.5%, *n* = 2) reported results indicating poor nutritional quality, but community cafes and education sessions had a positive impact on diet quality for participants post‐intervention. It is interesting to note that the paper by Frost et al. (2016) suggested one potential explanation for the poor nutritional quality of charity meals. According to the authors, this could be because volunteers who prepare or serve the food resist following healthy eating guidance due to their ethos of over‐provision (i.e., prioritising giving large/generous portions of comforting or familiar foods rather than its nutritional quality).

Fruit and vegetable schemes and educational interventions generally increased participants' fruit and vegetable intakes, but the magnitude of these increases varied considerably. This review found similar evidence reporting the impact of food insecurity interventions on diet and/or nutritional quality to other reviews (Blake and Cromwell 2022; Holley and Mason 2019; Loopstra 2018; Morris and Hitchcock 2022; Oldroyd et al. 2022). It is widely acknowledged that high intakes of saturated fat, sugar and salt that were identified by the foodbank studies are linked to preventable physical health conditions commonly associated with food insecurity, including hypertension (Stuff et al. 2004), diabetes (Seligman et al. 2007; Seligman et al. 2010) and hyperlipidaemia (Seligman et al. 2010). Poor quality diets are also linked to several mental health conditions, including anxiety and depression (The Mental Health Foundation 2022). Whilst the Food Ladders rung one interventions provide a lifeline for people in food insecurity crises, they are not a long‐term solution to food insecurity and may increase or exacerbate individuals' risk of preventable physical health conditions and/or mental health conditions. Rung two interventions showed more promise in improving participants' diet quality and provided participants with more accessible choice through shared activity around food (Blake 2019a). There was no evidence of the impact that rung three interventions had on diet or nutritional quality as this was not measured in the rung three studies (*n* = 2) (Mead et al. 2022; The Company Shop Group 2022). Some charities have started to consider the nutritional quality of food parcels they provide. One UK charity, CenterPoint, employs a team of Registered Dietitians that developed nutritional guidance for food parcels. This covers a range of ages and includes people with diabetes and those following a vegan diet (Centrepoint 2023). Furthermore, a blog post by Feeding America (2024) provides advice on suitable foods to donate that are of high nutritional quality. However, the impact of these nutritional guidelines on the nutritional quality of food provided has not been evaluated to the best of our knowledge.

Only one cross‐sectional impact report indicated post‐intervention reductions in food insecurity status (The Bread and Butter Thing 2022). Due to the methodological quality of this impact report being unknown, it is difficult to formulate any conclusions on the food insecurity impact. Furthermore, this report was a cross‐sectional design preventing the true impact of the intervention on food insecurity status from being measured (it was merely participant reported reductions in foodbank use or running out of food provided during a survey). This finding concurs with the evidence from similar existing systematic or scoping reviews that also found very few studies reporting on the impact on food insecurity status. However, these existing reviews reported that some interventions did appear to reduce food insecurity (Blake and Cromwell 2022; Holley and Mason 2019; Loopstra 2018; Morris and Hitchcock 2022; Oldroyd et al. 2022). The reason why the authors of studies included in our review did not use food insecurity as an outcome measure is unknown. One potential explanation is the lack of a UK‐validated food insecurity screening tool, although several international tools have been used previously in UK‐based surveys (Evidence and Network on UK Household Food Insecurity 2022), including those from the United States Department of Agriculture Food Security modules for adults (U.S. Department of Agriculture 2012a) and households (U.S. Department of Agriculture 2012b).

This review found that the elements for a successful intervention appear to be increasing knowledge, skills, and confidence; an ability to cater for special dietary, cultural and/or religious needs; providing additional financial/savings and/or debt management advice; removing barriers created by referral systems and processes; and providing space to socialise in the local community. These findings concur with the similar reviews (Blake and Cromwell 2022; Holley and Mason 2019; Loopstra 2018; Morris and Hitchcock 2022; Oldroyd et al. 2022).

### 4.2 Strengths and Limitations

To the best of our knowledge, this is the first UK review to map community‐based food insecurity interventions specifically targeted towards adults, investigate individual elements of interventions, and consider the outcomes in detail. Searches included extensive grey literature searches, forward and backward searches of included papers and any excluded reviews or commentary pieces. The main limitation of this review is the scarcity of literature, particularly peer‐reviewed literature regarding community‐based food insecurity interventions for adults in the United Kingdom and especially literature relating to process evaluations of interventions. Further limitations include the causality of the results due to the high proportion of cross‐sectional studies using convenience sampling, and the wide range of methodologies, variables and measurement tools used in included studies. Furthermore, many studies involved self‐reported outcome measures. Another limitation is that searches were limited to the United Kingdom only, and therefore the results are not generalisable outside of the United Kingdom. Finally, included studies were not critically appraised due to the limited quantity and scope of sources identified. Moreover, the detailed methodology for the conference abstracts and some impact reports was not described by authors, which prevented any appraisal of the quality of this review's included evidence. The methodological quality of the included studies is therefore unknown and may reduce the validity of the findings.

### 4.3 Recommendations for Future Practice, Policy and Research

There are several practice recommendations arising from this review. Firstly, evidence‐based standards for the nutritional quality of emergency food parcels and charitable meals are urgently required in the United Kingdom. Whilst one UK organisation, Centrepoint, has developed such guidance, its impact on the nutritional quality of emergency food should be evaluated through high‐quality research. Future intervention development should focus on increasing people's knowledge, skills and confidence to cook healthy meals on a budget; secure community spaces to socialise; have non‐mandatory referral systems; provide training for staff and volunteers regarding special dietary requirements, religious and cultural needs; and offer additional services such as financial and debt advice. As this review identified a heavy reliance on volunteers and donated food for intervention delivery, future policy should take a system‐wide approach to tackling food insecurity rather than over‐reliance on the voluntary and community sectors, and the goodwill of volunteers and donors. Due to the limited scope of available evidence on community‐based food insecurity interventions for UK adults, further peer‐reviewed research in this field is required, particularly relating to the rung two and three interventions on the Food Ladders. This research should include robust methodology; have a large and diverse sample; assess the impact of food insecurity interventions on food insecurity using a validated tool; and develop an agreed measure of diet quality for food insecurity interventions to fully understand their impact on diet quality. In particular, there is a need for more feasibility and acceptability studies to encourage a shift from volunteer‐driven interventions based on goodwill towards fully commissioned food insecurity services.

### 4.4 Main Conclusions

This review identified a very limited scope of evidence on community food insecurity interventions for UK adults, with the majority of peer‐reviewed studies focusing on emergency food provision. Over half of interventions relied on volunteers, and a high proportion used donated food. The nutritional quality of emergency food provision was reported to be poor, and special dietary, cultural and/or religious needs were not met. There were very few studies assessing the feasibility or acceptability of interventions for end users or those delivering interventions or evaluating their impact on reducing food insecurity. Elements that appeared important for a feasible and acceptable intervention were an educational approach to increase knowledge, skills and confidence; the ability to meet special dietary, cultural and/or religious needs; space in the community to socialise; removing barriers created by referral systems; and the provision of additional services such as financial or debt advice. Further research is required into the feasibility, acceptability and impact of interventions on reducing food insecurity for adults in the United Kingdom.

## Author Contributions

The author contributions are as follows: lead author (J.S.), senior academic support (E.L.G., A.A.L. and S.B.T.), concept (J.S., E.L.G., A.A.L. and S.B.T.), design of protocol and scoping review (J.S., E.L.G., A.A.L. and S.B.T.), research governance (J.S. and E.L.G.) data collection (J.S., P.H. and C.W.), data analysis (J.S., P.H. and C.W.) data management (J.S.), project management (J.S. and E.L.G.) drafting paper (J.S.), reviewing paper (E.L.G., A.A.L., S.B.T., P.H. and C.W.).

## Conflicts of Interest

The authors declare no conflicts of interest.

## Supporting information


**Table S1:** Preferred Reporting Items for Systematic reviews and Meta Analyses extension for Scoping Reviews (PRISMA‐ScR) Checklist.
**Table S2:** Keyword table.
**Table S3:** Hierarchy of Exclusion Table (for full article screen).

## Data Availability

Data sharing not applicable to this article as no datasets were generated or analysed during the current study.
